# How exercise influences the brain: a neuroscience perspective

**DOI:** 10.3325/cmj.2015.56.169

**Published:** 2015-04

**Authors:** Lukasz M. Konopka

**Affiliations:** Senior Executive Director of Chicago Brain Institute, Rolling Meadows, Il, USA

Today people expect advanced state-of-the-art diagnostic tools such as imaging and genomics to enhance their health and well-being, while simultaneously neglecting and ignoring their body’s physiology and what it requires for healthy development. An example testifying to this fact is the emerging increase in childhood and adult obesity across the globe (1). A factor contributing to this issue may be our belief that because technology makes us more efficient and productive, it gives us more time for a healthier active lifestyle; however, the opposite scenario seems true. The time and effort we might devote to physical activity, we replace with sedentary, passive activities that involve interacting with electronic media (2). We are so easily fascinated by acute informational access and the immediate rewards of computer games, web searches, and social media that they displace and distort our time substituting for the pleasure of physical activity.

Yet scientific data overwhelmingly argue that the human body was designed for physical activity that challenges our resting physiologic homeostasis. Throughout life, exercise causes measurable biological consequences that enhance well-being. Recent data show that exercise reduces the risk for many diseases such as breast and colon cancer, obesity, type-II diabetes, and cardiovascular disease, just to mention a few (3,4). Exercise was also shown to improve brain functions (5) . Current studies have demonstrated that cardiovascular exercise causes significant biochemical changes in the brains of animals. The biochemical molecule, brain derived growth factor (BDNF), causes the proliferation of neurons; vascular endothelial growth factor (VEGF) results in critical blood vessel growth; and an insulin-like growth factor (IGF-1) plays a significant role in exercise-induced angiogenesis—processes that are all enhanced by exercise (5,6). It is well-established that exercise increases perfusion, and angiogenesis directly increases the perfusion of the brain. Since the brain depends on oxygen and glucose, increased capillary beds enhance their delivery to brain tissue and, consequently, facilitate neuralplasticity (7). Neuroplasticity improves with the development of synaptic connections and neuronal networks. These structural and functional changes enhance learning and, ultimately, create a more efficient brain with a greater capacity to learn (8).

Increased neuroplasticity may be quite important. Studies have shown that at the time of crisis, cardiovascular exercise gives the brain a protective advantage by decreasing its vulnerability to vascular insults and protecting the blood brain barrier from oxidative stress (9,10). Recent studies have shown that patients who participated in aerobic fitness training had increased brain volumes and increased white and gray matter (11). The increased gray matter reflected increased numbers of neuronal cell bodies, whereas white matter areas showed increased axons (12). These findings correlated with functional studies of individuals who were in good cardiovascular condition. Additional electrophysiological studies showed that these individuals also had increased neuronal conduction velocity, which was related to white matter integrity, and increased response amplitudes, which correlated with cortical gray matter activation (13).

As we age, a well-functioning plastic brain becomes particularly important. Aging individuals in good physical health can expect enhanced cognitive reserves and a slower aging process (14). Even when patients begin to experience cognitive decline, the decline can improve using exercise as an intervention. For instance, patients with Alzheimer disease have shown significantly improved cognition and mood with exercise (15,16). Normal individuals and patients who suffer from psychiatric conditions such as attention deficit disorder, anxiety, and depression, respond positively to cardiovascular exercise (17). We now see that after cardiovascular exercise, children with attention deficit hyperactivity disorder (ADHD) improve on various measures such as attentional resource allocation, which may be directly related to how they modulate dopamine release (18). Patients with major depressive disorders rally following thirty minutes of moderate cardiovascular exercise and report significantly increased positive mood scores measured by mood states profile measures (19,20). Patients with anxiety seem to respond positively to acute cardiovascular exercise by endorsing fewer anxiety symptoms and a decreased probability of panic attacks (21,22). Their chronic anxiety symptoms are also reduced after aerobic exercise (23). Altogether, because the data indicate that exercise may provide significant effects, we should begin to incorporate exercise into our therapeutic designs for mood and cognitive disorders.

The role of exercise on brain function is well documented. Moreover, exercise is cheap, readily available, safe, and provides significant cognitive and emotional therapeutic effects. Therefore, it behooves us to incorporate exercise as part of the therapeutic armamentarium for medical and psychiatric disorders. The American College of Sports Medicine recommends that, optimally, healthy adults engage in more than thirty minutes of cardiovascular activity five days a week or twenty minutes of vigorous training three days a week. They also recommend resistance training that targets major muscle groups with activities such as balance, agility, coordination, and flexibility (24). Therefore, we must develop programs that educate our clinicians and the general population about the necessity of cardiovascular activity and the reduction of obesity in leading successful and productive lives.
